# Rare CNVs provide novel insights into the molecular basis of GH and IGF-1 insensitivity

**DOI:** 10.1530/EJE-20-0474

**Published:** 2020-09-17

**Authors:** Emily Cottrell, Claudia P Cabrera, Miho Ishida, Sumana Chatterjee, James Greening, Neil Wright, Artur Bossowski, Leo Dunkel, Asma Deeb, Iman Al Basiri, Stephen J Rose, Avril Mason, Susan Bint, Joo Wook Ahn, Vivian Hwa, Louise A Metherell, Gudrun E Moore, Helen L Storr

**Affiliations:** 1 Centre for Endocrinology, William Harvey Research Institute, Barts and the London School of Medicine & Dentistry, Queen Mary University of London, London, UK; 2 Centre for Translational Bioinformatics, Queen Mary University of London, London, UK; 3 NIHR Barts Cardiovascular Biomedical Research Centre, Barts and The London School of Medicine and Dentistry, Queen Mary University of London, London, UK; 4 University College London, Great Ormond Street Institute of Child Health, London, UK; 5 University Hospitals of Leicester NHS Trust, Leicester, UK; 6 The University of Sheffield Faculty of Medicine, Dentistry and Health, Sheffield, UK; 7 Department of Pediatrics, Endocrinology and Diabetes with a Cardiology Unit, Medical University of Bialystok, Bialystok, Poland; 8 Paediatric Endocrinology Department, Mafraq Hospital, Abu Dhabi, United Arab Emirates; 9 Mubarak Al-kabeer Hospital, Jabriya, Kuwait; 10 University Hospitals Birmingham NHS Foundation Trust, Birmingham, UK; 11 Royal Hospital for Children, Glasgow, UK; 12 Viapath, Guy’s Hospital, London, UK; 13 Addenbrookes Hospital, Cambridge, UK; 14 Cincinnati Center for Growth Disorders, Division of Endocrinology, Cincinnati Children’s Hospital Medical Center, Department of Pediatrics, University of Cincinnati College of Medicine, Cincinnati, Ohio, USA

## Abstract

**Objective:**

Copy number variation (CNV) has been associated with idiopathic short stature, small for gestational age and Silver-Russell syndrome (SRS). It has not been extensively investigated in growth hormone insensitivity (GHI; short stature, IGF-1 deficiency and normal/high GH) or previously in IGF-1 insensitivity (short stature, high/normal GH and IGF-1).

**Design and methods:**

Array comparative genomic hybridisation was performed with ~60 000 probe oligonucleotide array in GHI (*n* = 53) and IGF-1 insensitivity (*n* = 10) subjects. Published literature, mouse models, DECIPHER CNV tracks, growth associated GWAS loci and pathway enrichment analyses were used to identify key biological pathways/novel candidate growth genes within the CNV regions.

**Results:**

Both cohorts were enriched for class 3–5 CNVs (7/53 (13%) GHI and 3/10 (30%) IGF-1 insensitivity patients). Interestingly, 6/10 (60%) CNV subjects had diagnostic/associated clinical features of SRS. 5/10 subjects (50%) had CNVs previously reported in suspected SRS: 1q21 (*n* = 2), 12q14 (*n* = 1) deletions and Xp22 (*n* = 1), Xq26 (*n* = 1) duplications. A novel 15q11 deletion, previously associated with growth failure but not SRS/GHI was identified. Bioinformatic analysis identified 45 novel candidate growth genes, 15 being associated with growth in GWAS. The WNT canonical pathway was enriched in the GHI cohort and *CLOCK* was identified as an upstream regulator in the IGF-1 insensitivity cohorts.

**Conclusions:**

Our cohort was enriched for low frequency CNVs. Our study emphasises the importance of CNV testing in GHI and IGF-1 insensitivity patients, particularly GHI subjects with SRS features. Functional experimental evidence is now required to validate the novel candidate growth genes, interactions and biological pathways identified.

## Introduction

The growth hormone-insulin-like growth factor-1 (GH-IGF-1) axis is essential for normal human growth. Perturbations of this axis lead to growth hormone insensitivity (GHI, (MIM: 262500)) or IGF-1 insensitivity (MIM: 270450) (1). GHI is characterised by a triad of short stature (SS), functional IGF-1 deficiency and normal/high GH levels. Monogenic defects responsible for GHI have been identified in the growth hormone receptor (*GHR*) (2), *STAT5B* (3), *IGFALS* (4), *PAPPA2* (5) and *IGF1* (6) genes. Several distinct phenotypic features facilitate the clinical recognition of these specific genetic defects. IGF-1 receptor *(IGF1R)* mutations are characterised by IGF-1 insensitivity causing impaired foetal and postnatal growth associated with high/normal IGF-1 levels and microcephaly/developmental delay (7). A significant proportion of patients with clear evidence of GHI and IGF-1 insensitivity remains without a genetic diagnosis despite extensive investigation.

GHI-IGF-1 axis defects overlap with several other congenital syndromes such as Noonan syndrome (MIM: 163950), 3M syndrome (MIM: 273750) and Silver–Russell syndrome (SRS, (MIM: 180860)) (8). We previously reported two patients who presented with ‘classical’ GHI who were diagnosed with SRS secondary to 11p15LOM and upd(7)mat (8, 9). SRS is a clinical diagnosis. Characteristic features include being born small for gestational age (SGA), postnatal growth failure, relative macrocephaly at birth/protruding forehead, body asymmetry, feeding difficulty and/or low BMI. Diagnosis requires fulfilment of ≥4/6 Netchine–Harbison criteria (NH-CSS; including both prominent forehead and relative macrocephaly, termed ‘clinical SRS’), or 3/6 NH-CSS with a genetic defect recognised to cause SRS (10). The commonest underlying mechanisms are loss of methylation on chromosome 11p15 (11p15 LOM; 30–60% patients) and maternal uniparental disomy for chromosome 7 (upd(7)mat; 5–10% patients). The genetic aetiology is unknown in -40% ‘clinical’ SRS cases (11).

Other non-specific/associated SRS features include triangular face, low set ears, speech delay, high-pitched voice, micrognathia, down-turned mouth, low muscle mass, crowded/irregular teeth, clinodactyly and excessive sweating. These present in SRS children at higher frequency compared with non-syndromic/non-SRS SGA patients (10, 11). Some patients, particularly those with upd(7)mat, have fewer ‘typical’ clinical SRS features compared with 11p15 LOM individuals. Hence, SRS has wide clinical heterogeneity/variable severity and atypical presentations encompass the ‘SRS-like’ spectrum (12).

Advances in chromosomal microarray technologies have identified chromosomal imbalances across the human genome. These imbalances or copy number variations (CNVs), comprise deletions or duplications which can affect single or multiple genes or sections of chromosomes. CNVs encompass natural genetic variation and are often benign. However, rare CNVs are recognised to cause numerous complex traits such as autism, schizophrenia, Crohn’s disease and psoriasis and affect the susceptibility to HIV (13). The interpretation of potential disease-causing CNVs is challenging as they can exhibit variable penetrance and/or expressivity, that is, not all patients carrying the same CNV have the recognised disease phenotype or disease severity (14). Nevertheless, identification of CNVs is important as it may secure a diagnosis and facilitates the identification of key genomic regions and/or genes important for normal physiological processes.

Children with SS have a greater burden of rare CNVs (15, 16, 17) and a longer average CNV length compared to those with normal height (15, 16). Many have growth retardation in association with malformations and/or neurodevelopmental disorders (17). The incidence of pathogenic/likely pathogenic CNVs are reported as ~4–13% children with idiopathic SS, 16% born SGA with persistent SS and 14% with syndromic SS (15, 17, 18, 19, 20). Hence rare CNVs contribute to childhood SS and potential candidate genes and/or loci can potentially be identified (15, 17, 18, 19). More than 30 pathogenic CNVs have been identified in patients with suspected SRS (10, 11). Some have SRS-compatible phenotypes but many do not fulfil the NH-CSS criteria and frequently have more severe developmental delay and/or intellectual disability compared to ‘classic’ SRS patients (11). In one study, pathogenic CNVs were identified in 6% SRS-compatible patients and 7% SRS-like patients who were negative for classic SRS imprinting defects (21).

The identification of a pathogenic molecular defect is important for patients, families and clinicians as it avoids unnecessary investigations and/or treatment, ends uncertainty and allows appropriate genetic counselling. It is also fundamental to identify co-morbidities associated with syndromic SS. We investigated the role of CNVs in the aetiology of undiagnosed GHI and IGF-1 insensitivity and interrogated the genomic regions for novel candidate genes and pathways.

## Subjects and methods

### Ethical approval

Informed written consent for genetic research and publication of clinical details was obtained from patients and/or their parents. Approved by the Health Research Authority, East of England Cambridge East Research Ethics Committee (REC reference: 17/EE/0178).

### Subjects

**Figure 1:**
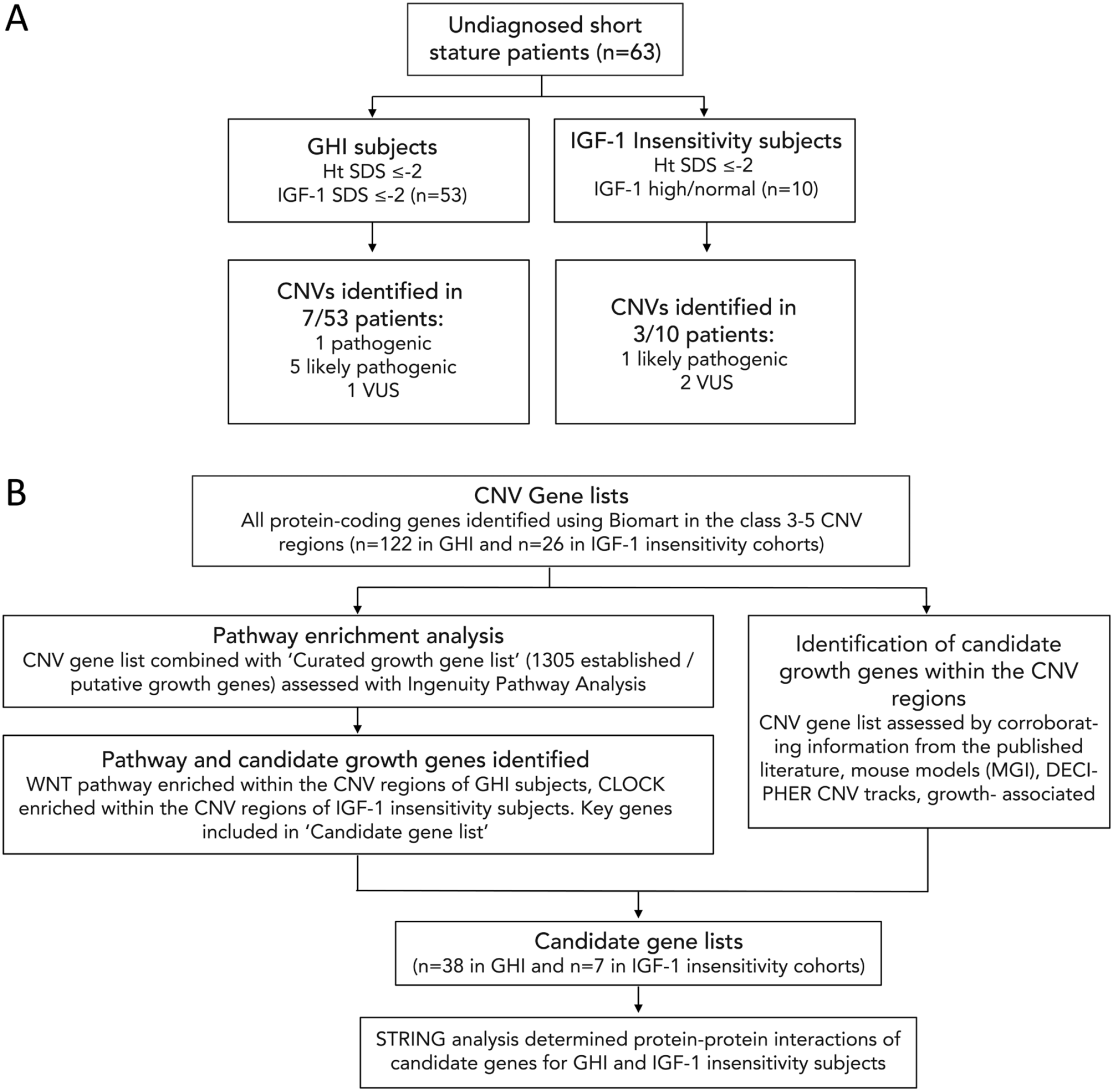
CNVs identified in the GHI and IGF-1 insensitivity subjects and bioinformatic pipeline used to identify key pathways and candidate growth genes in the CNV regions (A) CNVs identified in the GHI and IGF-1 insensitivity subjects. The most deleterious CNVs detected in each patient are listed. One patient with GHI and two patients with IGF-1 insensitivity had more than one CNV, so the CNV predicted to be most deleterious is listed. GHI, growth hormone insensitivity; SDS, standarddeviation score, CNV, copy number variant; VUS, variant of uncertain significance. (B) Flowchart showing the bioinformatic pipeline used to identify key pathways and candidate growth genes within the class 3–5 CNV regions identified in the patients. Given the distinct phenotypes, the CNV regions identified in the GHI and IGF-1 insensitivity patients were assessed separately. CNV, copy number variant; GHI, growth hormone insensitivity; Biomart http://grch37.ensembl.org/biomart/; MGI, mouse genome informatics http://www.informatics.jax.org/; DECIPHER https://decipher.sanger.ac.uk/; GWAS catalogue, genome wide association studies catalogue https://www.ebi.ac.uk/gwas/; STRING database https://string-db.org/. Full ‘CNV gene lists’ Supplementary Table 1.

Subjects were referred to our centre for genetic analysis between 2008 and 2019 (Fig. 1A). They were investigated at their home institutions and referring physicians completed a proforma detailing the clinical/biochemical data. Referring clinicians excluded growth hormone deficiency (peak GH level of ≥7 μg/L following standard provocation testing (51/63; 81%) or baseline GH of ≥10 ng/mL (12/63; 19%)) and causes of secondary GHI/IGF-1 insensitivity for example, undernutrition. Birth weight, height and BMI were expressed as standard deviation scores (SDS) according to the appropriate national standards. IGF-1 was expressed as SDS based on age/sex ranges provided.

### Growth hormone insensitivity (GHI) subjects

The GHI cohort (*n* = 53) (36 males, mean age: 7.4 years; range: 0.5–17.0) included 34 undiagnosed subjects following sequencing of the *GHR*, *IGF1* and *IGFALS* genes (exons and exon/intron boundaries), the *GHR* 6ψ pseudoexon and/or whole exome sequencing (WES) (8) and 19 new referrals. All 53 were undiagnosed following assessment by our in-house custom short stature gene panel covering the entire genomic sequence of 64 genes of interest which included all genes recognised to cause GHI and IGF-1 insensitivity and overlapping syndromes (3M, Noonan and SRS). All had SS (mean height SDS: −3.9, range: −2.0 to −7.4), normal/high growth hormone levels and IGF-1 deficiency (mean IGF-1 SDS: −2.5; range: −2.0 to −4.1). IGF-1 generation tests were performed at the referring centres according to established protocols in 27/53 (51%) subjects and an increase in IGF-1 level of <15 ng/mL between the basal and peak values consistent with severe GH resistance was noted in 13/27 (48%). Mean birth weight (BW) SDS was −1.1 (median: −0.95; range: 1.1 to −4.8) and 9/53 (17%) GHI subjects were born SGA (BW SDS: <−2.0) (Fig. 1A). Additionally, the sibling of the proband 1a was included in the bioinformatic analysis as she shared her sibling’s CNV and GHI clinical phenotype (height SDS: −1.6, GH peak: 18 µg/L, IGF-1 SDS: −2.4; patient 1b). Where serum IGF-1 was undetectable (less than the lower limit of the assay; *n* = 15), we calculated the lowest possible, detectable SDS (range: −2.4 to −3.0) and assigned this value for the statistical analysis. In these patients, IGF-1 SDS is likely to underestimate the degree of GH insensitivity.

### IGF-1 insensitivity subjects

The IGF-1 insensitivity cohort (*n* = 10) (6 male; mean age: 5.8 years, range: 1.1–16.5) included six undiagnosed subjects following sequencing of the *IGF1R* and 3M Syndrome (*CUL7, CCDC8* and *OBSL1*) genes (exons and exon/intron boundaries) and/or WES (8) and four new referrals (Fig. 1A). All ten were undiagnosed following assessment by our gene panel (above). All had SS (mean height SDS: −3.4, median: −3.5; range: −2.0 to −4.5) and normal/high GH and IGF-1 levels. Mean BW SDS was −2.0 (median: −2.1; range: −0.3 to −3.8) and 6/10 (60%) were born SGA (BW SDS <−2.0).

### Array comparative genomic hybridisation (aCGH) testing

Genomic DNA was isolated from peripheral blood leukocytes (Qiagen DNeasy Kit) and aCGH testing was performed at an ISO15189 accredited genetics laboratory. DNA samples were analysed by aCGH, using a 60K oligonucleotide array (Agilent design 028469 or 085030) as previously described (22). Briefly, 1 µg DNA was labelled using CGH Labelling Kit for Oligo Arrays (Enzo Life Sciences, USA), labelled DNA was purified post-labelling using QIAquick PCR purification Kit (Qiagen), DNAs were applied to a 60K oligonucleotide array (Agilent) and hybridisation, washing and scanning were performed following the manufacturers’ protocols.

### Array CGH data analysis

Array CGH data analysis was undertaken as previously described. Fluorescence signal intensity analysis was performed using Feature Extraction software (Agilent). CNV detection was performed using Genomic Workbench software (Agilent) and the ADM-2 algorithm (threshold 6). Secondary analysis was performed using the ADM-1 algorithm (threshold 6) for detection of low-level mosaicism (22). Population polymorphisms were filtered out and each CNV was assessed for pathogenicity in the context of each subject’s phenotype following the Association for Clinical Genetic Science (ACGS) best practice guidelines (23). GRCh37 (http://grch37.ensembl.org/index.html) was the reference genome. Parental samples ascertained the parent of origin or confirmed *de novo* event*s*. The median resolution of our CGH array was 120 kb.Five GHI subjects were analysed at reduced resolution (1–10Mb) due to poor DNA quality. No significant CNVs were identified in these patients.

### CNV classification

CNVs were classified into five categories (class 1, benign; class 2, likely benign; class 3, variant of uncertain significance (VUS); class 4, likely pathogenic and class 5, pathogenic) in line with accepted best practice guidelines (23). This classification process is based on available evidence from publications and public/laboratory databases, integrating population, computational, functional and segregation data. A CNV would be classified as pathogenic/likely pathogenic if in vivo or in vitro functional work supported this, it was *de novo* or inherited from an affected parent, it was recognised to cause an analogous phenotype and was not seen in normal healthy populations. Conversely, CNVs were classified as benign/likely benign if they did not segregate with the phenotype, were recognised in normal healthy populations, there was no functional work to prove their pathogenicity or there was data supporting benign classification. Class 1 or 2 CNVs were discarded and only pathogenic, likely pathogenic or VUS were investigated.

### Assessment of genes within the CNV regions

Class 3–5 CNV regions were explored for key pathways and candidate growth genes using bioinformatic analysis techniques (Fig. 1B). The CNV gene list was derived using Biomart (http://grch37.ensembl.org/biomart/) and included all the protein- coding genes within the class 3–5 CNV regions identified in the subjects (Supplementary Table 1, see section on supplementary materials given at the end of this article). The CNV gene list was interrogated to identify potential candidate growth genes Candidate gene list.

UCSC genome browser enabled visualisation of DECIPHER data (http://genome.ucsc.edu/). DECIPHER genome data tracks identified overlapping CNVs previously reported in growth failure subjects and hence key regions within the CNVs in our subjects. Mouse Genome Informatics (MGI) identified genes within the CNV gene list associated with pre- or postnatal growth failure phenotypes (http://www.informatics.jax.org/). Literature searches, Online Mendelian Inheritance in Man (OMIM, https://www.omim.org/) and pathway analyses also determined established and putative growth genes within the CNV regions. The NHGRI-EBI catalogue of published genome-wide association studies (https://www.ebi.ac.uk/gwas/) identified genes from the CNV gene list with loci associated with ‘height’.

### Pathway enrichment analysis

Ingenuity Pathway Analysis (IPA) software (Qiagen, Inc) identified biological pathways and functions enriched in the CNV regions (CNV gene lists) in both cohorts (GHI or IGF-1 insensitivity). Each individual was subject to an independent pathway analysis. Pathways with evidence of enrichment in more than three subjects were investigated further.

To investigate the enriched pathways further and ascertain their role in growth, we created the ‘Curated growth gene list’ (1305 established and candidate growth genes generated from published data and in-house analysis) (8, 24). Overlying the curated growth gene list with the pathway results allowed detection of pathways harbouring growth-related genes.

### In-silico protein-protein interaction analysis using Candidate gene lists

The combined bioinformatic analysis (above) produced *Candidate gene lists* for the GHI and IGF-1 insensitivity subjects (*n* = 38 GHI, *n* = 7 IGF-1 insensitivity). STRING database (https://string-db.org/) explored protein-protein interactions between the candidate genes (Human). Default settings were applied with the exception of interaction sources, where text mining and neighbourhood were excluded. Direct interactions between two candidate genes via intermediate proteins were explored.

### Statistical analysis

Phenotypic predictors (height SDS, age, sex, BW SDS, IGF-1 SDS) associated with the identification of subjects with and without CNVs were compared using 2-tailed *t*-tests and logistic regression analysis. The frequency of deletions vs duplications, the presence of SRS features and the size of the CNVs between the GHI and IGF-1 insensitivity cohorts were analysed by Fisher’s exact *t* and Mann–Whitney *U-*tests, respectively.

## Results

### Clinical and biochemical features of the CNV subjects

CNVs were identified in a total of 10/63 (16%) subjects; 7/63 (11%) (6 GHI, 1 IGF-1 insensitivity) had pathogenic or likely pathogenic CNVs (class 4/5) and 3/63 (5%) (1 GHI and 2 IGF-1 insensitivity) VUS CNVs (class 3) (Fig. 1A and Table 1). There were no significant differences between mean height, age, sex, BW SDS or IGF-1 SDS in the subjects harbouring CNVs compared to those without CNVs.

**Table 1:** Clinical and biochemical features of the patients harbouring CNVs. Patients 1a and 1b are siblings (1a is the proband included in the cohort, 1b exhibited the same GHI phenotype as her brother and harboured the same CNV). SS panel, custom gene panel covering entire genomic sequence of 64 genes associated with GH-IGF-1 axis defects causing GHI and IGF-1 insensitivity and overlapping short stature syndromes (SRS, Noonan and 3M).

Patient	Age at referral (years)	Sex	BW SDS	Height SDS	IGF-1 SDS	Previous genetic testing	Additional features
GHI subjects							
1a	3.8	M	−1.6	−3.6	−2.0	CGS, WES, SS panel, SRS**	Autistic spectrum
1b	1.1	F	−1.7	−1.6	−2.4	CGS, WES, SS panel	Language delay, dyslexia, recurrent ear infections
2	9.1	M	−0.4	−3.7	−2.3	CGS, WES, SS panel	–
3	11.3	M	−1.9	−5.1	−2.7*	CGS, WES, SS panel	–
4	1.9	M	−3.2	−5.7	−2.4	CGS, WES, SS panel, SRS**	Delayed motor development
5	17.0	M	−0.3	−4.0	−2.1	CGS, WES, SS panel	Delayed puberty, learning difficulties
6	2.8	M	−0.7	−4.9	−2.8	CGS, SS panel, SRS**	Persistent abdominal distention, bloating, severe constipation.
7	12.4	F	0.3	−2.5	−2.7	SS panel	Migraine, normal brain MRI
IGF-1 insensitivity subjects							
8	14.4	M	−2.2	−2.7	−0.6	CGS, SS panel	–
9	2.7	M	−2.1	−2.0	−0.8	CGS, SS panel	Adrenal insufficiency
10	2.5	F	−1.3	−3.6	1.3	SS panel	–

*IGF-1 undetectable on assay. **Negative testing for 11p15 LOM ± upd(7)mat undertaken at the referring centre. Patients 1–7, 9 and 10 are included in the previous publication (8).

BW, birth weight; CGS, candidate gene sequencing (GHR, GHR 6ψ, IGFALS and IGF1 for GHI group and 3M syndrome genes, CUL7, CCDC8, OBSL1 and IGF1R for IGF-1 insensitivity group) ; F, female; GHI, growth hormone insensitivity; M, male; NK, not known; WES, whole exome sequencing.

### CNVs in the GHI and IGF-1 insensitivity subjects

Class 3–5 CNVs were identified in 7/53 (13%) subjects with GHI (6 males; mean height SDS: −4.2, range: −2.5 to −5.7). 6/53 (11%) patients (5 males; mean height SDS: −4.3) had pathogenic or likely pathogenic CNVs (class 4/5). We identified CNVs classes 3–5 in 3/10 (30%) subjects with IGF-1 insensitivity (2 males, mean height SDS: −2.8, range: −2.0 to −5.7). One of these patients had a CNV classified as likely pathogenic. Details of CNV subjects in Supplementary results.

### SRS features in the CNV subjects

Interestingly, five GHI patients with class 3–5 CNVs had clinical features of SRS (NH-CSS ≥2 in addition to SRS-like features, Table 2). Patient 2 fulfilled the NH-CSS criteria (3/6 with a recognised genetic cause; 1q21 deletion). A total of 6/7 (86%) GHI subjects harbouring CNVs had SRS features or recognised associated features (Table 2) in contrast to 8/46 (17%) GHI subjects without CNVS, suggesting CNVs were much more likely to be present in GHI subjects with SRS features/associated features (*P* < 0.0001). Five patients (4 GHI) had CNVs previously reported in suspected SRS: 1q21 (*n* = 2), 12q14 (*n* = 1) deletions and Xp22 (*n* = 1) and Xq26 duplications (*n* = 1).

**Table 2:** SRS features in the patients harbouring CNVs. Patients 1a and 1b are siblings (1a is the proband included in the cohort, 1b exhibited the same GHI phenotype as her brother and harboured the same CNV). NH-CSS, Netchine−Harbison SRS clinical scoring system: diagnosis of SRS requires fulfilment of 4/6 (including both prominent forehead and relative macrocephaly, termed ‘Clinical SRS’) or 3/6 in addition to a genetic diagnosis associated with SRS. The criteria are: (a) SGA (birth weight and/or birth length ≤−2 SDS for gestational age); (b) Postnatal growth failure (height at 24 ± 1 months ≤−2 SDS or height ≤−2 SDS below mid-parental target height); (c) Relative macrocephaly at birth (head circumference at birth ≥1.5 SDS above birth weight and/or length SDS); (d) Protruding forehead (forehead projecting beyond the facial plane on a side view at 1–3 years); (e) Body asymmetry (leg length discrepancy (LLD) of ≥0.5 cm or arm asymmetry or LLD <0.5 cm with at least two other asymmetrical body parts, one non-face); (f) Feeding difficulties and/or low BMI (BMI ≤−2 SDS at 24 months or use of feeding tube or cyproheptadine appetite stimulant) (10).

Patient	CNV	NH-CSS criteria	Additional SRS features*	CNV previously associated with short stature/SRS
GHI subjects				
1a	1q21 deletion	2 (b,f)	Triangular face, high arched palate, hypoglycaemia, clinodactyly	SRS features (43) and 1q21 deletion syndrome (MIM: 612474)
1b	1q21 deletion	1 (f)	Speech delay	SRS features (43) and 1q21 deletion syndrome (MIM: 612474)
2	1q21 deletion	3 (b,c,f)	Clinodactyly	SRS features (43) and 1q21 deletion syndrome (MIM: 612474)
3	12q14 deletion	2 (b,f)	Triangular face, high pitched voice	SRS features (50) and 12q14 deletion syndrome** (30)
4	7q21 deletion, 7q31 deletion	2 (a,b)	Triangular face, low set ears, delayed motor development	None reported
5	5q12 deletion	1 (b)	Nil	5q12 deletion syndrome, (MIM: 615668) (37)
6	15q11 deletion	3 (b,d,f)	Triangular face, hypoglycaemia	15q11 deletion, (MIM: 615656)
7	Xq26 duplication	1 (b)	Brachydactyly, downturned mouth	SRS features (43)***
IGF-1 insensitivity subjects				
8	7q21 duplication, Xp22 duplication	2 (a,b)	Nil	SRS features with Xp22 duplication (36). Xp22 duplication also identified in SGA cohort (17).****
9	7q36 duplication	2 (a,b)	Nil	None reported
10	3p22 deletion, 15q13 duplication	1 (b)	Nil	None reported

*Additional clinical features recognised in SRS (11); **No OMIM number assigned to this syndrome currently;***The duplication described in Spengler *et al.* begins at Xq25 (genomic co-ordinates 129 132 238-139 650 444) whilst our patient duplication begins at Xq26 (co-ordinates 134 842 275-141 407 613); ****Both these duplications described in the literature are larger than the CNV identified in our patient and encompass the SHOX region and/or the SHOX enhancer region, whilst our CNV does not include either.

### Details of the CNVs identified in the GHI and IGF-1 insensitivity subjects

A total of 13 class 3–5 CNVs were identified in 10 subjects; 5/13 (38%) CNVs have previously been reported in SRS-like patients (Table 3). 7/10 subjects had genomic deletions** (143 487–9 111 383 bp). In total, 4/10 subjects had genomic duplications (283 862–6 565 338 bp). Two of the duplications were found in association with another deletion or duplication (patients 8 and 10, respectively). Two deletions were identified in patient 4. Two unrelated patients were found to have 1q21 deletions (patients 1a and 2). Patient 1a’s sibling with a similar phenotype harboured the same CNV and was included in the bioinformatic analysis (patient 1b). A total of 122 and 26 protein-coding genes reside in the CNV regions of the GHI and IGF-1 insensitivity cohorts, respectively (Supplementary Table 1).

**Table 3:** Details of CNVs identified in our patients.

Patient	CNV^1^	Class^2^	Inheritance	Parental height SDS	Size (Mb)	Number of affected protein- coding genes	DECIPHER^3^	Mouse genome database^4^	Key candidate gene(s) in this region^5^
GHI subjects									
1a	1q21.1q21.2(146564742_147735011)x1	4	Maternal	+0.08	1.17	9	26	3	*BCL9b,d,e, PRKAB2c,d, FMO5c,d, GPR89Bc, CHD1Ld,e*
1b	1q21.1q21.2(146641600_147735011)x1	4	Maternal	+0.08	1.09	9	26	3	*BCL9b,d,e, PRKAB2c,d, FMO5c,d, GPR89Bc, CHD1Ld,e*
2	1q21.1q21.2(145987155_147735011)x1	4	*De novo*		1.74	11	26	3	*BCL9b,d,e, PRKAB2c,d, FMO5c,d, GPR89Bc, CHD1Ld,e*
3	12q14.2q15(64413681_67794677)x1	5	NK		3.38	21	9	4	*HMGA2a,c,d, WIF1b, XPOTc, IRAK3c, GRIP1c , LLPH d, MSRB3d, SRGAP1d*
4	7q21.2(91914300_92762100)x1	3	*De novo*		0.85	9	4	3	*ANKIB1 c,d, PEX1 c,d, CDK6 c,d, SAMD9d,e, GATAD1d*
	7q31.1q31.31(111130598_120241981)x1	4	*De novo*		9.11	29	7	8	*WNT2 b,c, IMMP2Lb, c, IFRD1c, GPR85 c, FOXP2 c,d, CAV1 c, MET c, CFTR c, CAV2d PPP1R3Ad, TFECd*
5	5q12.1q12.2(60371468_62950178)x1	3	NK		2.58	10	3	2	*ZSWIM6c, CKS1Bc,e, DIMT1e*
6	15q11.2(22765627_23085096)x1	4	NK		0.32	4	29	0	-
7	Xq26.3q27.2(134842275_141407613)x3	4	*De novo*		6.57	38	14	6	*SOX3a,b, ZIC3c, BRS3 c, RBMX c, CD40LGc, FHL1c*
IGF1 insensitivity subjects									
8	7q21.13(89733373_90035738)x3	3	Maternal	–1.67	0.30	5	1	0	–
	Xp22.33(1793445_2213992)x3	3	Paternal	−0.41	0.42	1	8	0	–
9	7q36.1(151748853_152032715)x3	3	Maternal	+0.33	0.28	2	3	1	*GALNT11b, KMT2Cc*
10	3p22.1(41611009_41754496)x1	3	Maternal	−0.75	0.14	1	2	1	*ULK4c*
	15q13.2q13.3(30653877_32914199)x3	4	Paternal	−3.35	2.26	17	14	2	*CHRNA7b, KLF13b, FAN1c, OTUD7Ac*

1a (proband) and 1b are siblings with shared GHI phenotypes. ^1^Co-ordinates given are relative to version 37 of the human genome. ^2^CNV class 3, variant uncertain significance (VUS); 4, likely pathogenic; 5, pathogenic (50). ^3^Number of patients in DECIPHER with overlapping deletion/duplication (as per patient CNV) and pre/postnatal growth restriction. ^4^Number of genes causing a growth restricted phenotype in the mouse model (Mouse Genome Informatics http://www.informatics.jax.org/). ^5^Candidate genes were identified by corroborating information from current literature, MGI, DECIPHER, GWAS database information and our bioinformatic pathway analysis. Candidate genes in the CNV regions were selected as they were: ^a^established in literature to have important role in normal linear growth;^ b^identified as candidate gene from our bioinformatic pathway analysis; ^c^causing a growth restricted phenotype in the mouse model; ^d^Containing loci associated with height in GWAS catalogue (https://www.ebi.ac.uk/gwas/) or in GWAS literature; ^e^putative growth role based on current literature. A total of 38 candidate genes were identified in the CNV regions of our GHI cohort and seven genes in the CNV regions of our IGF-1 Insensitivity cohort.

Except for one outlier in each cohort, the CNVs in the GHI subjects were significantly larger (>500 000 bp) than those identified in the IGF-1 insensitivity subjects (<500 000 bp) (*P* = 0.03). All but one CNV identified in the GHI subjects were deletions and all but one CNV in the IGF-1 insensitivity cohort were duplications (not significant, likely due to the small sample size).

### Pathway enrichment analysis

**Figure 2:**
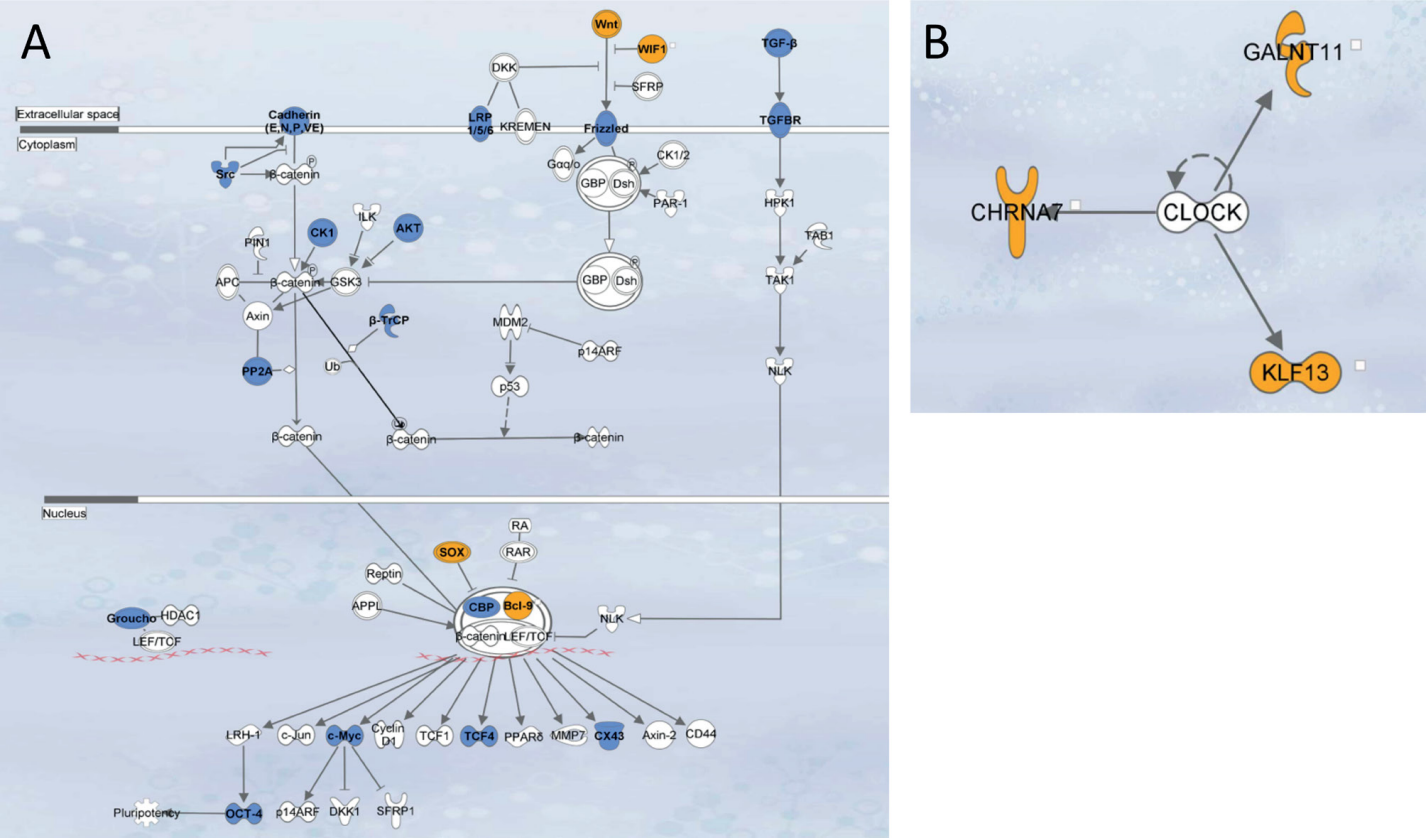
Pathway enrichment analysis (A) Identification of the WNT pathway enriched within the gene list from CNV regions of the growth hormone insensitivity (GHI) subjects. Genes highlighted in blue are from our curated growth candidate gene list of 1305 genes. Yellow genes are those genes found within in the CNV regions (*WIF1* in the 12q14 deleted region of patient 3*, WNT2* in the 7q31 deletion of patient 4, *SOX3* in the Xq26 duplicated region of patient 7 and *BCL9* in the 1q21 deleted region of patients 1a, 1b and 2). (B) Pathway enrichment analysis identified CLOCK as a transcription regulator enriched within the CNV gene list of the IGF-1 insensitivity subjects. Genes highlighted in yellow reside in our patient’s CNV regions. CLOCK is a transcription regulator for genes *GALNT11*, *CHRNA7* and *KLF13* within the CNV regions. *GALNT11* lies within the 7q36 region duplicated in patient 9 and both *KLF13* and *CHRNA7* are found within the 15q13 region duplicated in patient 10.

Ingenuity pathway analysis (IPA) (Qiagen, Inc) identified pathways and biological functions enriched within the CNV regions of individuals in the same cohort (GHI or IGF-1 insensitivity). The WNT canonical pathway was enriched in two GHI individuals (patients 1–2) and patient 1b (the sibling of patient 1a) (Benjamini-Hochberg adjusted *P*-value=0.11). Additionally, WNT pathway genes were observed in three other GHI subjects whose enrichment evidence was weak (patients 3, 4 and 7) (Fig. 2A). IPA upstream analysis of IGF-1 insensitivity subjects identified CLOCK as a plausible common upstream regulator in two IGF-1 insensitivity patients (Fig. 2B).

### Identification of candidate genes

**Figure 3:**
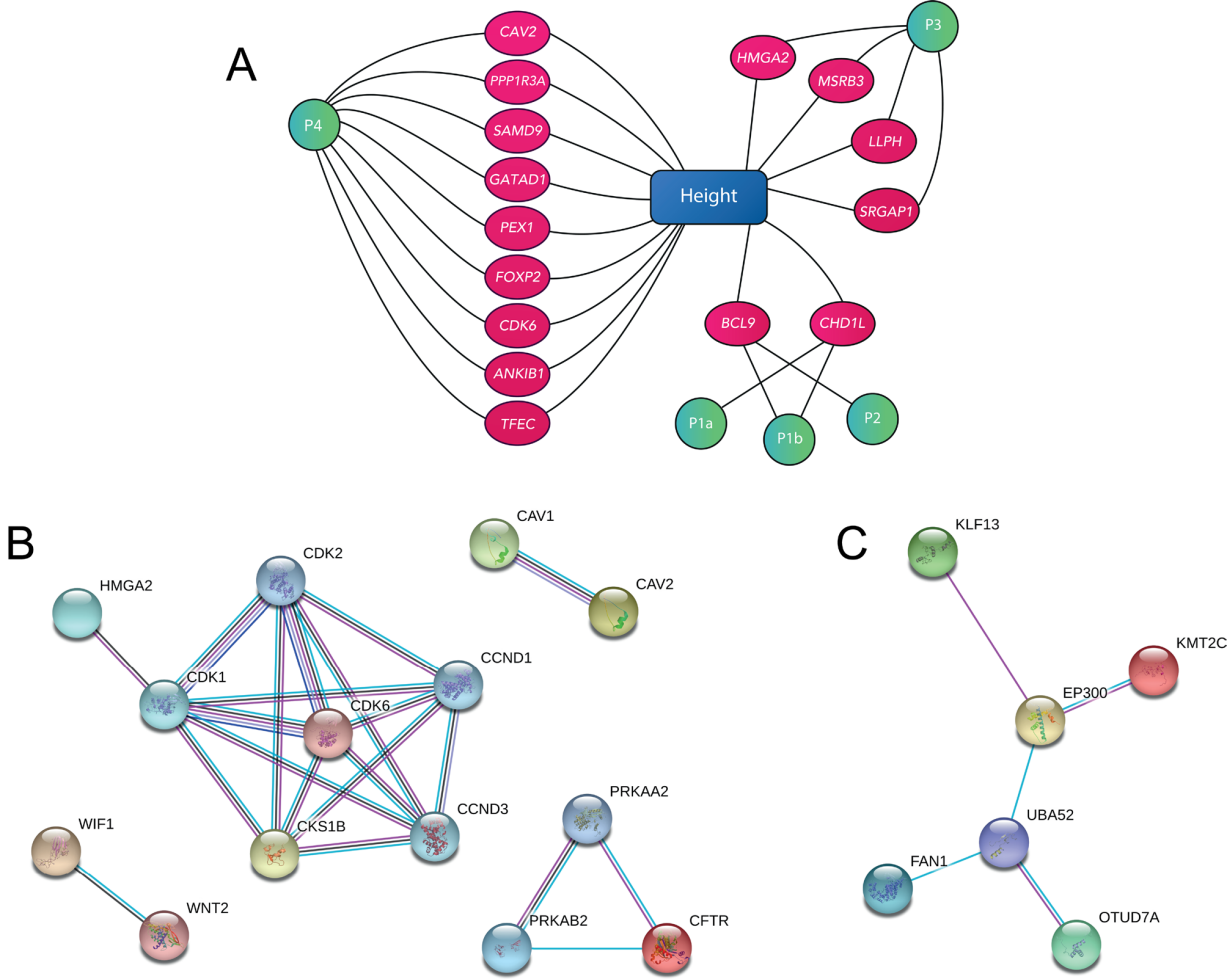
GWAS and STRING analysis of genes within the CNV regions (A) Genes in our patient’s CNVs associated with height in the GWAS catalogue. Genes are shown in the blue circles and are connected to the patient(s) (P) in which the CNV containing that gene was identified. The NHGRI–-EBI catalogue of published genome-wide association studies (https://www.ebi.ac.uk/gwas/) was used to examine all protein-coding genes within the class 3–5 CNV regions in the subjects (CNV gene lists). Those CNV genes that have loci with associations to height were identified. Protein-protein interactions of the candidate genes in the CNV regions of (B) The growth hormone insensitivity (GHI) cohort (38 genes). STRING analysis (https://string-db.org/) identified abundant protein-protein interactions (direct and indirect via intermediate proteins) between genes in the CNV regions (candidate gene list). Default settings were used with the exception of interaction sources, where text mining and neighbourhood sources were excluded. Cyclin-dependent kinase 6 (CDK6, patient 4) and cyclin-dependent kinases regulatory subunit 1B (CKS1B, patient 5) interact directly and also with high mobility group AT-Hook 2 (HMGA2, patient 3) via cyclin-dependent kinase 1 (CDK1). Cyclin-dependent kinase 2 (CDK2), Cyclin D1 (CCND1) and cyclin D3 (CCND3) also interacted with both CDK6 (patient 4) and CKS1B (patient 5). Direct interaction was identified between Wnt inhibitory factor 1 (WIF1, patient 3) and Wnt family member 2 (WNT2, patient 4). Protein kinase AMP-activated non-catalytic subunit B2 (PRKAB2, patients 1a, 1b and 2) and cystic fibrosis transmembrane conductance regulator (CFTR, patient 4) interacted directly and also via 5’-AMP-activated protein kinase catalytic subunit alpha-2 (PRKAA2). Caveolin-1 (CAV1) and caveolin-2 (CAV2) directly interact and both were identified in patient 4’s CNV (C) IGF-1 insensitivity cohort (7 genes). Kruppel-like factor 13 (KLF13, patient 10) interacts with lysine methyltransferase 2C (KMT2C, patient 9) via E1A-associated protein p300 (EP300). Additionally, OUT domain-containing protein 7A (OTUD7A, patient 10) and fanconi-associated nuclease 1 (FAN1, patient 10) interacts with EP300 via ubiquitin A-52 residue ribosomal protein fusion product 1 (UBA52).

Forty-five candidate growth genes were identified within the CNV regions of 9/10 (82%) subjects (Table 3). Fifteen of these genes in five subjects were associated with height in the NHGRI-EBI catalogue of published genome-wide association studies (Fig. 3B). Some CNV regions harboured strong candidate growth genes for example, *HMGA2* (12q14 deletion; patient 3). In other regions, candidate genes were proposed based on their roles in known growth pathways/mouse models. The limitation is that only protein-coding candidate genes are identified by this approach and not imprinting control regions which may impact growth for example, chromosome 7 alterations.

### *In silico* protein–protein interaction analysis of candidate gene list

STRING analysis identified abundant protein–protein interactions (direct and indirect) between genes in the CNV regions (candidate gene list) of the GHI cohort (38 genes) and the IGF-1 insensitivity cohort (7 genes) (Fig. 3B and C). Interactions were identified between several proteins involved in regulating cell cycle progression: CDK6, CKS1B, HMGA2, CDK1, CDK2, CCND1 and CCND3. Overrepresentation of cyclin dependent protein kinases in CNV loci suggests an association between these genes and short stature.

## Discussion

GHI is typically associated with classic Laron syndrome causing severe SS, dysmorphic and metabolic abnormalities. Advancing genetic techniques increasingly recognise a spectrum of genetic defects responsible for mild-moderate GHI phenotypes including ‘non-classic’ GH receptor defects such as dominant-negative heterozygous variants, the *GHR* pseudoexon and other GH-IGF-1 signalling pathway defects (25). Our findings suggest that CNVs contribute to the genetic aetiology of undiagnosed non-classical GHI patients. Haploinsufficiency or duplication of gene(s) in the affected region(s) may be sufficient to affect growth but not as severely as total loss of function/homozygous mutations seen in ‘classic’ GHI.

DNA microarrays detect imbalances in chromosomal regions (chromosomal microarray analysis, CMA). Two CMA techniques detect chromosomal imbalance, comparative genomic hybridization (CGH) and SNP. CGH‐based arrays (aCGH) measure the quantity of genomic DNA in a patient’s sample compared with a ‘normal’ (aged and sex matched) control. Typical clinical CGH arrays contain several hundred thousand probes but research CGH arrays may incorporate millions. SNP arrays use DNA probes from genomic regions and reveal differences between individuals at a single bp site. As well as copy number data, SNP arrays can detect other clinically important features for example, uniparental disomy (UPD). Most SNP arrays used for clinical purposes are hybrid arrays containing both SNP and copy number probes some with some having in excess of 2.6 million probes. A higher probe concentration will detect smaller imbalances (microdeletion/duplications).

We previously noted the overlap of GHI with SRS (8, 9). Interestingly, almost half of the GHI patients harbouring CNVs had subtle clinical features of SRS and one patient fulfilled the NH-CSS criteria (10). The NH-CSS criteria are well-defined and are highly sensitive (~98%) for detecting classic 11p15 LOM/matUPD. As such, patients who do not fulfil the criteria are unlikely to have either of the two classic molecular defects (10). However, as with many clinical assessment tools, there is a degree of subjectivity and the speciﬁcity is low (36%). Therefore, NH-CSS may not be appropriate to detect patients with milder SRS-like disorders due to other genetic abnormalities. The SRS consensus recognises >30 different CNVs in patients with suspected SRS. Many do not fulfil the NH-CSS criteria and are more frequently associated with learning difficulties than classic SRS. The current recommendation is to manage patients according to their CNVs rather than SRS (11). Whilst none of the GHI subjects with CNVs had learning difficulties, the milder clinical phenotypes overlap with the SRS-like patients with CNVs. This is interesting as 40% clinical SRS patients have no genetic diagnosis, and exploring CNV regions in detail may enable the discovery of novel genes and pathways responsible for SRS-like phenotypes and other childhood growth disorders (11). Our findings also expand the SRS-like spectrum and may lead to further stratification of SRS subtypes.

Forty per cent (4/10) of the patients in our cohort inherited their CNV from a parent, 75% (3/4) with normal heights. This could imply that the CNVs identified are not or are only partially responsible for the observed growth failure in these patients. However, none of the subjects had any other potential causative genetic variants identified following assessment by our custom short stature gene panel which included whole genomic regions for 64 genes recognised or suspected to cause similar phenotypes. It is also conceivable that their growth failure is attributable or partially attributable to as yet unidentified variants elsewhere in the genome, abnormalities in methylation or other genetic aberrations.

Variable penetrance is a recognised feature of many CNVs and can cause a significant challenge in assessing the pathogenicity of CNVs. Several modifiers may account for this, including the background genetic variation of individuals and/or epigenetic mechanisms such as imprinting, expression or regulatory variation of the genes in the affected CNV region. It is also possible that recessive variants residing on the single remaining allele are unmasked in some individuals (26). Interestingly, we identified 1q21 deletions in two unrelated GHI patients and a sibling of a proband. 1q21 deletion syndrome is recognised to cause dysmorphism, learning difficulties, growth failure in 50% and autism (MIM: 612474). Dysmorphic features include microcephaly, frontal bossing, deep-set eyes, epicanthal folds, large nasal bridge, long philtrum and high-arched palate. Most 1q21 deletions are inherited in an autosomal dominant manner and exhibit variable penetrance, with ‘affected’ parents frequently having no or very mild phenotype (27). Analysis of 4737 unaffected individuals did not identify any 1q21.1 deletions, suggesting the CNV is responsible for the phenotype (27). Potential causes of the wide phenotypic variability have not been delineated. Analysis of genes within the affected regions of eleven 1q21 deletion carriers did not support unmasking of recessive variants as a cause of the variable phenotypes. Data from an affected 1q21 deletion patient and her unaffected carrier mother, suggested that differences in methylation of the non-deleted 1q21.1 locus did not contribute to the phenotypic variability. Parent-of-origin studies show both maternal and paternal transmission of the deletion, therefore it is unlikely that imprinting plays a role (27).

Potential genes in the 1q21 region responsible for growth failure are not established. We identified several candidate genes within the commonly deleted region including chromodomain-helicase-DNA-binding protein 1-like* (CHD1L). CHD1L* regulates chromatin relaxation/cell cycle progression and knockdown in glioma cells result in reduced proliferation (28). B-cell CLL/lymphoma 9 protein* (BCL9)* is a co-activator of Wnt/β-catenin signalling, an evolutionarily conserved signalling pathway fundamental for embryonic development, tissue homeostasis, cellular proliferation and growth (29). Loci in *BCL9* and *CHD1L* were also associated with height in the GWAS catalogue (*P*-values: 7.00E-13 and 7E-20, respectively). 5’-AMP-activated protein kinase* (PRKAB2)* and flavin containing monooxygenase 5* (FMO5)* also reside in genomic loci associated with height in genome-wide association studies (24) and have essential roles in cellular energy and glucose homeostasis.

Patient 3 had a 12q14 deletion and there is strong evidence to suggest that haploinsufficiency of high-mobility group AT-hook 2* (HMGA2)* causes the observed growth failure in patients with deletions in this region (30). Additionally, submicroscopic 12q14 deletions, spanning only part of *HMGA2,* have been reported in three individuals with SS and SRS features (31). A study examining three *HMGA2* single-nucleotide polymorphisms (SNPs) in 155 idiopathic SS patients and 318 normal stature controls concluded they contributed to short stature susceptibility (32). Heterozygous *HMGA2* point mutations have been identified in two subjects with SS and SRS-like features, causing a frameshift and a premature stop codon, respectively (33). A heterozygous 7bp intronic deletion causing aberrant splicing of *HMGA2* has also been described in a patient with a similar phenotype (34). *HMGA2* is thought to function as an upstream regulator of IGF2 but the mechanism is not characterised or understood (33). Loci in *HMGA2, LLPH, MSRB3* and *SRGAP1* were reported in the GWAS catalogue to be associated with height with minimum *P*-values of 1.00E−287, 3.00E−21, 4.00E−39, 3.00E−12, respectively.

Patients 4 and 8 had CNVs affecting 7q21 and one additional region. Both fulfilled 2/6 NH-CSS (SGA with postnatal growth failure). SRS patients with matUPD7 defects have milder phenotypes compared with ICR1 hypomethylation and less asymmetry (35). Patient 4 had 7q21 and 7q31 deletions with associated SRS features (triangular face, low set ears and delayed motor development). Both deletions are adjacent to the 7q21.3 (PEG10/SGCE) and 7q32 (MEST) imprinting clusters. Several genes within the CNV regions of patient 5 had GWAS height loci: *TFEC* (*P*-value: 5.00E−07), *ANKIB1* (*P*-value: 2.00E−15), *CDK6* (*P*-value: 3.00E−240), *FOXP2* (*P*-value; 2.00E−15), *PEX1* (*P*-value: 1.00E−08), *GATAD1* (*P*-value: 1.00E−08), *SAMD9* (2.00E−25), *PPP1R3A* (*P*-value: 2.00E−08) and *CAV2* (*P*-value: 1.00E−11). Patient 8 had duplications of 7q21 and Xp22. Xp22 duplications have been identified in SRS and SGA subjects and included SHOX and a SHOX enhancer region (PAR3-12), respectively (17, 36). The CNV identified in our subject does not encompass either of these regions and both known duplications were larger than the CNV identified in our patient suggesting the mechanism in our subject is either independent of SHOX function or does not contribute to the phenotype.

Patient 5 had a 5q12 deletion. 5q12 deletion syndrome has been reported in several patients with postnatal growth failure and developmental delay (37). This region harbours two candidate genes. Cyclin-dependent kinases regulatory subunit 1 *(CKS1B)* and dimethyladenosine transferase 1 homolog gene *(DIMT1). CKS1B* has an essential role in cell cycle regulation. Cks1-depleted breast cancer cells exhibit slow G1 cell cycle progression and G2-M arrest due to blocked mitotic entry (38). *DIMT1* is a methyltransferase essential for ribosome biogenesis and overexpressed in several cancers (39). The functional roles of these genes are likely to be critical for normal linear growth and haploinsufficiency may lead to postnatal growth failure.

Prader–Willi (PWS) and Angelman syndrome are classically caused by chromosome 15 deletions of different parental origin involving the distal breakpoint BP3 and proximally placed breakpoints BP1 or BP2 at 15q11 (40). Patient 6 had a small 15q11 deletion encompassing BP1 and BP2. 15q11.2 deletions (including just BP1 and BP2) have variable phenotypes and expressivity (40). Short stature and unspecified dysmorphic features are reported in 10 and 39% patients, respectively. Although not a reported feature of 15q11.2 deletions, our patient had significant feeding difficulties. Interestingly, feeding problems are a common feature of PWS.

Patient 7 was the only GHI subject with a duplication. The Xq26.3q27.2 duplicated region harbours SRY-related HMG-box 3 *(SOX3)* encoding a transcription factor implicated in embryonic development regulation. *SOX3* under- and over-expression in males causes multiple pituitary hormone, isolated growth hormone deficiency associated with infundibular hypoplasia, ectopic/undescended posterior pituitary and abnormalities of the corpus callosum with or without intellectual disability (41). Our patient had normal brain MRI and sufficient GH secretion but low IGF-1 (SDS −2.7). A female patient is reported with poor growth and low IGF-1. The authors suggest her poor growth is due to GH deficiency, but formal GH stimulation testing was not performed (42). Another female patient with a larger duplication is reported in a cohort of patients with SRS features. Interestingly, this patient had a broad nasal bridge similar to patient 8, with triangular face and relative macrocephaly. The published duplication begins at Xq25 (genomic co-ordinates: 129 132 238–139 650 444) and in our subject at Xq26 (co-ordinates; 134 842 275–141 407 613) (43). The overlapping region includes *SOX3* and several other genes.

Chromosome 7q36 rearrangements are very rare, with deletions being more prevalent than duplications. Seventy per cent 7q36 deletion individuals exhibit growth retardation (44), however, few duplications have been reported. One patient with a larger 7q36 duplication than patient 9 was born SGA with no catch-up growth, developmental delay and multiple congenital abnormalities including a cardiovascular malformation, sensorineural hearing loss, myopia, astigmatism, cryptorchidism, hypospadias, microphallus and dysmorphic facial features (45). One interesting candidate gene, *GALNT11,* is common to both duplicated regions. *GALNT11* encodes polypeptide N-acetylgalactosaminyltransferase 11, which O-glycosylates NOTCH1 and activates Notch signalling (46). Abnormal *GALNT11* dosage (reduced or enhanced) may alter Notch signalling and adversely affect growth. Patient 9’s duplication was inherited from his mother who is of normal stature. This suggests variable penetrance (similar to 1q21 deletions) or that this CNV is not responsible for the observed phenotype.

A 3p22 deletion and 15q13 duplication were identified in patient 10. These CNVs have not previously been associated with short stature or SRS. Interestingly, the 15q13 duplication was inherited from her father who has significant short stature (height SDS −3.4). Within this region lies several candidate genes. Both *FAN1* and *OTUD7A* cause a growth restricted phenotype in mouse models, whilst *CHRNA7* and* KLF13* were identified by ingenuity pathway analysis (IPA).

IPA identified genes with links to established or novel growth pathways in the CNV regions of our subjects. Six GHI individuals had CNVs harbouring genes in the canonical WNT pathway. *WIF1* in the 12q14 deleted region of patient 3*, WNT2* in the 7q31 deletion of patient 4, *SOX3* in the Xq26 duplicated region of patient 7 and *BCL9* in the 1q21 deleted region of patients 1a, 1b and 2. The WNT pathway determines cell fate and is a key regulator of cell proliferation (47). Aberrant Wnt activity leads to uncontrolled cell growth and oncogenesis and is a potential novel therapeutic target for cancer. Abnormal copy numbers of these genes may impair this pathway and normal linear growth. The 12q14 deletion identified in patient 3 includes *WIF1* and *HMGA2*. The latter is thought to be the most crucial growth regulating gene in this region; however it is possible both contribute important growth regulatory effects.

CLOCK regulates the transcription of *GALNT11*, *CHRNA7* and *KLF13* genes residing within the CNV regions identified in the IGF-1 insensitivity subjects. This is particularly interesting as IGF-1 regulates clock gene expression and functions as a zeitgeber for cellular hypothalamic circadian rhythms (48). In addition to its central functional role in the regulation of circadian rhythm, CLOCK modulates G2-to-M cell cycle transition facilitating cell cycle progression and proliferation (49).

In summary, this is the first study investigating in detail CNVs in subjects with growth failure associated with GHI and IGF-1 insensitivity. Rare CNVs were a relatively common cause of milder non-classical phenotypes in our cohort. This expands the known phenotypes of rare CNVs, potentially identifies new candidate SS genes and expands the spectrum of GHI/IGF-1 insensitivity and overlapping disorders such as SRS. CNV analysis should be carried out in all short patients where no monogenic cause has been identified, particularly those with features of GHI, IGF-1 insensitivity and concomitant subtle features of SRS. Functional experimental evidence is now required to validate the candidate growth genes, interactions and biological pathways enriched in our cohort.

## Supplementary Material

Supplementary Results Details of the CNVs identified in the GHI and IGF-1 insensitivity subjects (Table 3)

Supplementary Table 1. Complete list of protein-coding genes within each CNV region ‘CNV gene lists’

## Declaration of interest

The authors declare that there is no conflict of interest that could be perceived as prejudicing the impartiality of this study.

## Funding

This work was supported by a Barts Charity Large Project Grant (Grant Reference Number: MRC0161) awarded to HLS and the 2018 European Society for Paediatric Endocrinologyhttp://dx.doi.org/10.13039/100010381 (ESPE) Research Fellowship awarded to E C.

## Author contribution statement

E C conducted the study and wrote the manuscript. H L S conceptualised/designed the study, wrote and approved the final version of the manuscript. C P C conducted and interpreted the bioinformatic analysis. S C undertook the statistical analysis. M I and G E M were involved in conception of study and critically revised aspects of manuscript. L A M and V H gave intellectual input and revised drafts of the manuscript. J G, N W,A B, L D, A D, I A B, S R and A M acquired essential clinical and biochemical data. S B, J W A undertook CNV analysis and provided expert interpretation of data.
