# Supplementary material for: Rare CNVs provide novel insights into the molecular basis of GH and IGF-1 insensitivity
Source: Eur J Endocrinol. 2020 Sep 17;183(6):581–95. doi: 10.1530/EJE-20-0474 (PMC7592635; doi:10.1530/EJE-20-0474)
Supplement: Supplementary Results Details of the CNVs identified in the GHI and IGF-1 insensitivity subjects (Table 3) [file supplementary_material.pdf]

Supplementary Results

Details of the CNVs identified in the GHI and IGF-1 insensitivity subjects (Table 3)

CNVs in the GHI subjects

Two patients in our GHI cohort had 1q21 deletions (patient 1a and 2). The sibling of patient 1a (patient 1b; from a non-consanguineous family) was also found to carry the deletion. The proband (patient 1a) had height SDS -3.6 with a small triangular face and high arched palate and BW SDS -1.6. SRS testing (11p15 LOM and upd(7)mat) at the referring centre was negative. The younger sibling (patient 1b) had height SDS -1.6 and BW SDS -1.7 with no dysmorphic features. Both had feeding problems in infancy and recurrent infections. The older sibling experienced hypoglycaemic episodes and was also diagnosed with autistic spectrum disorder. The younger sibling had dyslexia. The deletion was inherited from their mother, whose height SDS was 0.1. Patient 2 had no dysmorphic features, height -3.8 SDS and BW SDS -0.4. This patient was the offspring of non-consanguineous parents who had normal phenotypes and the deletion was *de novo*.

A 12q14 deletion was identified in patient 3, a with BW SDS -1.9, height SDS -5.1, triangular face and high-pitched voice. This subject required nasogastric feeding for poor weight gain. Interestingly, there was an older sibling with similar facial features and poor growth and two half siblings, who also had growth failure. Patient 4 had two *de novo* deletions on chromosome 7 (7q21 and 7q31). This patient was born SGA (BW SDS -3.2) and had triangular face, low set ears and delayed motor development. SRS testing (11p15 LOM and upd(7)mat) at the referring centre was negative.

A 5q12 deletion was identified in patient 5, a with postnatal growth failure (height SDS -4.0), delayed puberty and learning difficulties. Patient 6 had a 319Kb deletion of material from within chromosome 15q11.2, flanked by BP1 and BP2 of the Prader-Willi syndrome/Angelman syndrome

region. This patient had postnatal growth failure (height SDS -4.9), relative macrocephaly, triangular face, hypoglycaemic episodes and feeding difficulties. The subject had low BMI (-2.0 SDS) and received supplemental enteral feeds and suffered with persistent abdominal distention, bloating and severe constipation but intestinal biopsies were unremarkable. SRS testing (11p15 LOM and upd(7)mat) at the referring centre was negative.

A *de novo* Xq26.3-q27.2 duplication was detected in patient 7, a 12-year-old patient with height SDS -2.5, brachydactyly, a downturned mouth and a broad nasal bridge.

CNVs in the IGF-1 insensitivity patients

Two CNVs were detected in patient 8, a 14-year-old who was born SGA (BW SDS -2.19) with poor catch-up growth (height SDS -2.7): array CGH identified a maternally inherited 7q21 duplication and a paternally inherited Xp22 duplication. Both parents had normal stature (height SDS -1.67 and -0.41, respectively).

A 7q36 duplication was identified in a 2-year-old (patient 9) with adrenal insufficiency who was born SGA (BW -2.05) with no catch-up growth. A 3p22 deletion and 15q13 duplication were identified in patient 10, a 2-year-old patient with postnatal growth failure (height SDS -3.6).
