# Supplementary material for: Rare CNVs provide novel insights into the molecular basis of GH and IGF-1 insensitivity
Source: Eur J Endocrinol. 2020 Sep 17;183(6):581–95. doi: 10.1530/EJE-20-0474 (PMC7592635; doi:10.1530/EJE-20-0474)
Supplement: Supplementary Table 1. Complete list of protein-coding genes within each CNV region ‘CNV gene lists’ [file supplementary_table_1.pdf]

Supplementary Table 1. Complete list of protein-coding genes within each CNV region 'CNV gene lists'

Patient	CNV	Genes in the CNV region(s)
**Growth Hormone Insensitivity (GHI) subjects**		
1a	1q21 deletion	*PRKAB2, FMO5, CHD1L, BCL9, ACP6, GJA5, GJA8, GPR89B, NBPF24*
1b	1q21 deletion	*PRKAB2, FMO5, CHD1L, BCL9, ACP6, GJA5, GJA8, GPR89B, NBPF24*
2	1q21 deletion	*NBPF11, NBPF12, PRKAB2, FMO5, CHD1L, BCL9, ACP6, GJA5, GJA8, GPR89B, NBPF24*
3	12q14 deletion	*SRGAP1, C12orf66, C12orf56, XPOT, TBK1, RASSF3, AC025262.1, GNS, TBC1D30, WIF1, LEMD3, MSRB3, HMGA2, RP11-366L20.2, AC090673.2, LLPH, TMBIM4, IRAK3, HELB, GRIP1, CAND1*
4	7q21 deletion<br><br>7q31 deletion	*ANKIB1, GATAD1, ERVW-1, PEX1, RBM48, FAM133B, CDK6, SAMD9, SAMD9L*<br><br>*IMMP2L, DOCK4, ZNF277, IFRD1, LSMEM1, TMEM168, C7orf60, GPR85, LINC00998, TSRM, PPP1R3A, FOXP2, MDFIC, TFEC, TES, CAV2, CAV1, MET, CAPZA2, ST7, ST7-OT4, WNT2, ASZ1, CFTR, CTTNBP2, NAA38, ANKRD7, KCND2*
5	5q12 deletion	*NDUFAF2, AC008498.1, SMIM15, ZSWIM6, C5orf64, KIF2A, DIMT1, IPO11, CKS1B, LRRC70*
6	15q11 deletion	*TUBGCP5, CYFIP1, NIPA2, NIPA1*
7	Xq26 duplication	*CT45A1, CT45A2, CT45A3, CT45A4, CT45A5, CT45A6, SAGE1, MMGT1, SLC9A6, FHL1, MAP7D3, GPR112, BRS3, HTATSF1, VGLL1, CD40LG, ARHGEF6, RBMX, GPR101, ZIC3, FGF13, F9, MCF2, ATP11C, CXorf66,*

		SOX3, LINC00632, CDR1, SPANXB2, SPANXB1, LDOC1, SPANXC, SPANXA1, SPANXA2, SPANXD, MAGEC3, MAGEC1, MAGEC2
**IGF-1 Insensitivity subjects**		
8	7q21 duplication<br><br>Xp22 duplication	*STEAP1, STEAP2, C7orf63, GTPBP10, CLDN12*<br><br>*DHRSX*
9	7q36 duplication	*GALNT11, KMT2C*
10	3p22 deletion<br><br>15q13 duplication	*ULK4*<br><br>*CHRFAM7A, GOLGA8R, GOLGA8Q, GOLGA8H, ARHGAP11B, FAN1, MTMR10, TRPM1, KLF13, OTUD7A, CHRNA7, GOLGA8K, AC139426.2, GOLGA8O, AC135983.2, GOLGA8N, ARHGAP11A*

CNV, Copy Number Variant; IGF-1, Insulin-like growth factor 1.
